# Starving in the land of plenty: the challenge of studying menstruation in anticoagulated patients

**DOI:** 10.1016/j.rpth.2024.102500

**Published:** 2024-06-29

**Authors:** Bethany T. Samuelson Bannow

**Affiliations:** ^1^The Hemostasis and Thrombosis Center, Oregon Health & Science University, Portland, Oregon, USA; ^2^Division of Hematology & Medical Oncology, Department of Medicine at OHSU, Knight Cancer Institute, Oregon Health & Science University, Portland, Oregon, USA

Menstruation affects half the global population for roughly 40 years of life. Up to a third of these individuals will experience heavy or otherwise abnormal uterine bleeding. Among those individuals who require anticoagulation for venous thromboembolism (VTE) or other indications, the rate of heavy menstrual bleeding (HMB) skyrockets to 70% [1]. While many treatment options are available for HMB, few have been studied in anticoagulated patients, and prescribers often feel confused about or uncomfortable with these strategies.

The MEDEA study [2] was a randomized, open-label, pragmatic clinical trial designed to compare 2 treatment strategies: (1) switch from a factor (F)Xa inhibitor to dabigatran, a direct thrombin inhibitor, and (2) addition of tranexamic acid (TXA), an antifibrinolytic, during menses while continuing the FXa inhibitor to no treatment (control arm) for the management of HMB in the setting of anti-Xa therapy. Each participant was randomized to 1 of these 3 arms with the intent of comparing Pictorial Blood Loss Assessment Chart (PBAC) scores from prior to randomization (T1) with those from the 3 subsequent menstrual cycles on the intervention. Regretfully, the study was terminated early due to low enrollment and thus was not adequately powered to detect the differences it sought to identify. Based upon data collected from the 16 randomized individuals, however, Hamulyák et al. [2] report they were able to detect a statistically significant decrease in PBAC scores before and after initiation of TXA (−199; 95% CI, −343 to −54). The team also identified a nonsignificant decrease in PBAC scores with a switch to dabigatran and a slight, nonsignificant increase in those who continued anti-Xa therapy without intervention.

While it is disappointing that the study was forced to close early, the study team must be applauded for recognizing the significant gaps in research for HMB, which affects an alarming proportion of individuals requiring anticoagulation, and for making a commendable effort to ascertain the impact of 2 potential treatment strategies in this unique population. Although underpowered, the findings are meaningful and contribute important work to the growing research field of HMB.

In non–anticoagulated patients, effective therapies for HMB include TXA, combined hormonal contraceptives, and progestin-only hormonal therapies, including the levonorgestrel intrauterine device (Figure). Patients who do not experience an adequate response to these therapies or who have additional complications or indications may also be considered for procedural therapies such as endometrial ablation, uterine artery embolization, or hysterectomy.

**Figure:**
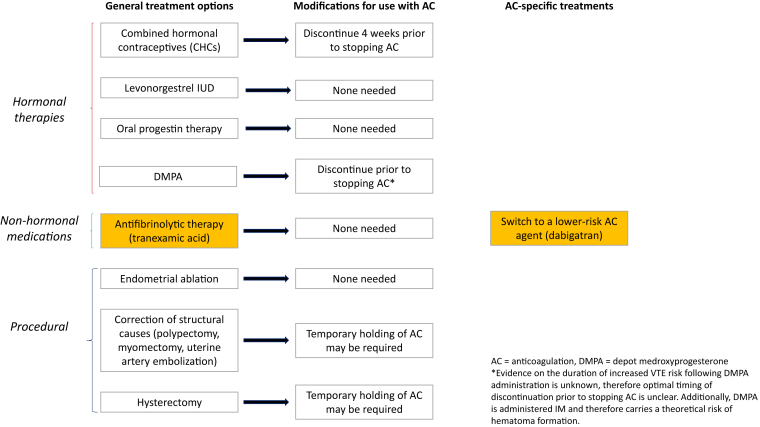
Management strategies for abnormal uterine bleeding, including modifications for patients requiring anticoagulation and therapies relevant only to those on anticoagulation. AC, anticoagulation; CHC, combined hormonal contraceptive; DMPA, depot medroxyprogesterone acetate; IUD, intrauterine device. ∗Evidence on the duration of increased venous thromboembolism risk following DMPA administration is unknown; therefore, the optimal timing of discontinuation prior to stopping AC is unclear. Additionally, DMPA is administered intramuscularly and therefore carries a theoretical risk of hematoma formation.

Although available data suggest that therapeutic rivaroxaban (20 mg daily) may be associated with numerically increased menstrual flow duration and intensity compared with prophylactic dosing (10 mg daily), dose reduction has not demonstrated statistically significant differences in menstrual bleeding patterns [3]. More importantly, the risk of VTE recurrence can be expected to increase when anticoagulation is withheld or, perhaps, even in the setting of early or inappropriate dose reduction, as supported by evidence of increased risk of VTE recurrence in individuals with HMB on rivaroxaban [4]. Thus, it was appropriate that the authors did not include this strategy as an additional arm in MEDEA.

While these various strategies can be, and often are, used in the treatment of anticoagulated individuals with HMB, data specifically comparing safety and efficacy of treatments in this population are lacking. Furthermore, despite evidence suggesting safety when used in therapeutically anticoagulated individuals [5], discontinuation of combined hormonal contraceptives is often a knee-jerk reaction when a patient is diagnosed with VTE. This can lead to a recurrence of HMB, worsened in the setting of anticoagulation. Due to widespread but demonstrably false assumptions that TXA increases risk of VTE [6], patients may also be denied this essential therapy. Thus, inclusion of this treatment option in the MEDEA study was important and, hopefully, precedent-setting despite the inability to complete enrollment as planned.

The rationale for switching from an anti-Xa inhibitor to dabigatran for management of anticoagulant-associated HMB is based upon post hoc analyses of large, randomized controlled trials of direct oral anticoagulants, which included some menstruating individuals. This analysis demonstrated decreased rates (odds ratio, 0.59) of abnormal uterine bleeding in individuals on dabigatran compared with low-molecular-weight heparin/warfarin. It is worth noting, however, that the rates of warfarin-associated HMB were higher in the dabigatran trials than in trials of the Xa inhibitors. This fact, in combination with the potential challenges with real-world anticoagulant switching, such as payor coverage restrictions, suggests that addition of TXA may be the more practical of the 2 interventions investigated in MEDEA.

The fact that this first randomized, interventional study of treatment options for an adverse effect impacting up to 70% of reproductive-aged female users of these anticoagulants comes more than 15 years after approval of the first direct oral anticoagulants highlights a major limitation with current data and methodology of anticoagulant studies. Studies of anticoagulants to date have not been designed to detect HMB as an adverse effect and thus the scope of the problem remains underappreciated, and data on direct management are absent.

The authors of this study used a PBAC to make the diagnosis of HMB. This is a perfectly reasonable and rather straightforward option that involves participant completion of a form that documenting number of menstrual products used and degree of saturation. This method is much more accessible and less costly than the “gold standard” alkaline hematin method, which involves collection of menstrual products and spectrophotometric quantification of hemoglobin contained therein. Limitations of the PBAC include reliance on disposable pads and tampons and sometimes reliance on participant memory if the form is not filled out contemporaneously.

An alternative that may be considered, and which may or may not have resulted in different findings or higher rates of inclusion, is the Menstrual Bleeding Questionnaire (MBQ) [7]. In addition to objective data such as frequency of product saturation, the MBQ collects data on the impact of menstruation on quality of life. This is particularly relevant as the most up-to-date definition of HMB includes impact on physical, emotional, social, and material quality of life. In working with individuals with HMB, we have found that retrospective PBAC scores are almost always higher than contemporaneously collected ones, thus suggesting that the experience and impact of HMB are not fully captured by PBACs. Use of metrics such as the MBQ to determine inclusion may result in a lower rate of screen failures in future studies while still capturing essential outcomes.

Future studies of HMB in anticoagulated individuals may also benefit from more robust screening and recruitment methods. As described, the MEDEA study depended on identification of individuals with HMB by the treating physician. As pointed out by the authors themselves, many cases of HMB likely go undetected due to clinician and patient hesitancy or failure to bring up the topic. If discussion of menstruation and patient and clinician awareness of HMB were ubiquitous, there is little doubt that a much larger sample size could have been obtained.

Critically, early closure of the MEDEA study should not be interpreted as proof that future studies in this area cannot be successful. Despite being late in coming in comparison with the release of Xa inhibitors, the MEDEA study suffered from being ahead of its time in examining interventions for a problem that clinicians are not yet appropriately recognizing and diagnosing. Rather than disappointment over inadequate enrollment, the takeaway message of this study should be a call to action to improve our recognition of this common issue so that future studies may be better powered to provide the answers patients suffering from this condition need.
